# Comorbid Anxiety, Depression and PTSD in Tunisian Military Veterans

**DOI:** 10.1192/j.eurpsy.2025.1946

**Published:** 2025-08-26

**Authors:** Y. Ben Othmene, A. Baatout, K. Kefi, W. Kabtni, S. Eddif, H. Kefi, A. Oumaya

**Affiliations:** 1 Psychiatry, Military Hospital, Tunis, Tunisia

## Abstract

**Introduction:**

Intrusion, hyperarousal, avoiding triggers and alterations in cognition and mood are the symptoms defining a post-traumatic stress disorder (PTSD).

While PTSD can occur in individuals from all walks of life, its prevalence and severity are higher among military veterans, exacerbating the impact of other mental health disorders such as anxiety and depression and impairing quality of life.

**Objectives:**

The current study sought to determine the prevalence of comorbid anxiety and depression with PTSD among Tunisian military veterans.

**Methods:**

A cross-sectional descriptive and analytical survey was conducted between September and November 2024, focusing on Tunisian veterans seeking consult, using a data file and 2 self-report scales :

The PTSD Checklist for DSM-5 (PCL-5) to assess current PTSD symptoms with a cut-off score of 33 or higher to detect PTSD cases.

The Hospital Anxiety and Depression (HAD) scale, which consists of two subscales: the Anxiety (A) subscale and the Depression (D) subscale. For both subscales, scores are ranging from 0 to 21: [0-7]: normal, [8-10]: borderline case, [11-21]: an abnormal level of anxiety or depression.

To analyze the obtained data, IBM SPSS was used.

**Results:**

The study enrolled 24 veterans diagnosed with PTSD, with the majority being male (87.5%). Mean age of the participants was 34 [23-50] years. Most (58.3%) were married, 33.3% were single, 4.2% were divorced, and 4.2% were in a relationship.

A significant proportion of the surveyed (79.2%) were smokers, while 29.2% reported occasional alcohol consumption. None reported using illicit drugs, such as cannabis.

Regarding medical history, 29.2% had medical health conditions including asthma, diabetes, hypertension, herniated disc, and kidney stones.

In terms of psychiatric care, 95.8% were under regular psychiatric follow-up and 54.2% reported having a support system.

Regarding psychiatric comorbidities, 95.8% (N=23) of participants presented with anxiety symptoms with 4.2% falling into the borderline category. For depression, 66.7% reported depressive symptoms, 25% were classified as borderline cases and 8.3% showed no depressive symptoms.

Median PCL-5 score was 55.5±12.15. Half of the population (N=12) had a score higher than 55. All of them exhibited depressive symptoms. In contrast, among those with lower scores, only 33.3% had depressive symptoms, 50% had borderline cases and 16.7% had no depressive symptoms.

A significant correlation was found between **PCL-5** scores above 55 and the presence of depressive symptoms (p=0.02), suggesting a strong association between higher PTSD severity and depression in this sample.

**Conclusions:**

This study reveals a high prevalence of comorbid anxiety and depression among Tunisian Military veterans suffering from PTSD with a significant association between higher PTSD severity and depressive symptoms, highlighting the need for integrated mental health care that addresses both PTSD and its comorbidities.

**Disclosure of Interest:**

None Declared

